# Delayed Dentition

**Published:** 1888-09

**Authors:** W. J. Stevenson


### Delayed Dentition.

*Editor Medical World*:—In the *World* for April, page 138, I find a communication from Dr. J. B. Patton on "Delayed Dentition." We admit it to be a physiological process, and everything being equal, it is painless; but how often do we find it otherwise? The Doctor speaks of lancing the gums as cruel the indiscriminate use of the lancet, I confess, is cruel and does no good; in fact, harm is done if the tooth is delayed, as it then has to overcome the resistance of the cicatrix. But what does he do when he finds the little fellows with convulsions, gums tense and white from strangulation? Does he rely entirely on sedatives and improvement of the blood, until the offending member comes throngh? When I find the above, my lancet is the first thing I think of, and usually in a day or two the tooth makes its appearance. In but a few instances I have had to lance the same gum more than once. A few years ago I was called to see a child some 12 or 14 months old with convulsions; had several before my arrival; found gum over lower incisors tense and white; went down to the tooth with lancet, gave one dose of bromide of potasssium (about ten grs.) In a half hour my little patient was sleeping sweetly and the tooth came through in a day or two. After lancing I always direct the gums rubbed frequently with finger to prevent union. My ideas may be erroneous on the use of the lancet, and. if such should be the case, I am open to conviction.

Lauderdale, Miss. W. J. STEVENSON.

*Editor Medical World*: EVER SINCE THE subject has been agitated by the *World* I have abandoned those old graveyard sign-posts "ʒ" and "℥," and have written my prescriptions with "dr." and "oz." *They cannot be confounded by any sober man.* The *World* deserves the thanks of the world for a timely agitation.—G. V. Hale, M. D. (Jeff.) Wheatland, Texas.

WHEN A THIN MAN visits you, lodge him in the spare room, of course.—*Life.*

